# Differences between the Anæsthesia of Ether and Chloroform

**Published:** 1876-09

**Authors:** 


Differefnces between the Anæsthesia of Ether and Chloroform.—Prof. Schiff (*Brit. Med. Jour.* May 22, 1875) relates the results of more than five thousand experiments on the differences between ether and chloroform anæsthesia. With both paralysis of conscious sensation, paralysis of the movement of voluntary muscles, paralysis of respiration, circulation, and, finally, paralysis of the heart and the vaso-motor nerves occurs. Respiratory paralysis is produced by ether when circulation and blood pressure remain within the limits compatible with life. Sometimes the vascular pressure increases, sometimes it decreases, but is always sufficiently high to allow the exchange of the carbonic acid gas with the oxygen of the atmosphere. Vascular succeeds respiratory paralysis when ether is administered. The reverse takes place with chloroform. Frequently an amount of this anæsthetic agent which would not be sufficient to produce respiratory may suffice to bring on vascular paralysis. Under these conditions, and when vascular paralysis lasts under thirty seconds, artificial respiration is useless, because there is no longer any exchange of gases, the blood pressure being diminished. The cessation of respiration, therefore, is not the most dangerous moment to animal life, when etherization is employed, whilst it may be so with chloroformization, because sometimes it is possible to produce some automatic respiratory movements, but, nevertheless, respiration ceases immediately, and the animal dies. With etherization, on the contrary, when some automatic inspirations are obtained, it may be taken as certain that respiration will continue, and that the animal will live. Schiff affirms that in the present state of our science there are no means which will show us how to recognize, so as to prevent them, the tendencies which may cause death in some animals after the first inhalations of chloroform, before having produced true anæsthesia. The reverse occurs with ether, so that it may be said that, in the present state of knowledge, the surgeon is responsible for the death of the individual by etherization, whilst he is not responsible when death occurs from chloroformization. From these facts Schiff concludes:

1. The phenomena relating to the paralysis of sensibility and movement are common to both ether and chloroform.

2. The two other orders of phenomena—that is to say, those relating to vascular and respiratory paralysis—often show themselves in inverse order with reference to these two agents.

3. With chloroform, however, either the one or the other of these two paralyses may first show itself, involving great danger to the animal if the vascular phenomena be the first to make their appearance. Therefore the use of chloroform should be rejected, and ether only used.—*Journal of Materia Medica.*

